# Education of medical personnel optimizes filling volume of blood culture bottles without negatively affecting microbiology testing

**DOI:** 10.1186/s12913-020-05959-z

**Published:** 2020-12-01

**Authors:** Katrin Steiner, Joanna Baron-Stefaniak, Alexander M. Hirschl, Wolfgang Barousch, Birgit Willinger, David M. Baron

**Affiliations:** 1 Department of Anaesthesia, Intensive Care Medicine and Pain Medicine, Medical University of Vienna, Waehringer Guertel 18-20, 1090 Vienna, Austria; 2 Department of Laboratory Medicine, Division of Clinical Microbiology, Medical University of Vienna, Waehringer Guertel 18-20, 1090 Vienna, Austria

**Keywords:** Anemia, Blood culture testing, Blood loss, Educational activity, Patient blood management

## Abstract

**Background:**

Anemia is a risk factor for adverse outcomes, which can be aggravated by unnecessary phlebotomies. In blood culture testing, up to 30 ml of blood can be withdrawn per sample, even though most manufacturers recommend blood volumes of 10 ml or less. After assessing the filling volume of blood culture bottles at our institution, we investigated whether an educational intervention could optimize filling volume of blood culture bottles without negatively affecting microbiology testing.

**Methods:**

We weighed 10,147 blood cultures before and 11,806 blood cultures after a six-month educational intervention, during which employees were trained regarding correct filling volume via lectures, handouts, emails, and posters placed at strategic places.

**Results:**

Before the educational intervention, only 31% of aerobic and 34% of anaerobic blood cultures were filled correctly with 5–10 ml of blood. The educational intervention increased the percentage of correctly filled bottles to 43% (*P* < 0.001) for both aerobic and anaerobic samples without negatively affecting results of microbiologic testing. In addition, sample volume was reduced from 11.0 ± 6.5 to 9.4 ± 5.1 ml (*P* < 0.001) in aerobic bottles and from 10.1 ± 5.6 to 8.8 ± 4.8 ml (*P* < 0.001) in anaerobic bottles.

**Conclusion:**

Education of medical personnel is a simple and effective way to reduce iatrogenic blood loss and possibly moderate the extent of phlebotomy-induced anemia.

## Background

Anemia is common in critical illness, and has been identified as an independent risk factor for increased perioperative morbidity and mortality [1]. Treatment of anemia represents a central pillar of patient blood management, a concept which reduces red blood cell utilization [2–6]. Another important aspect of patient blood management is prevention of anemia by avoiding unnecessary blood sampling and blood loss [7, 8]. A simple measure to minimize blood loss and prevent phlebotomy-induced anemia is by using small-volume sampling tubes.

In blood culture testing, up to 30 ml of blood can be withdrawn with current systems, even though manufacturers recommend filling volumes of 10 ml or less. Thus, bottles have to be actively disconnected to prevent excess filling or alternatively have to be filled with syringes, a sampling technique associated with possible contamination and needle-stick injury [9]. Only limited data are available regarding the filling volume of blood culture bottles in a routine clinical setting [10]. Thus, the first aim of this study was to assess the filling volume of blood culture bottles at our institution.

Educational interventions are usually cost-efficient, and have positively influenced knowledge and performance of medical personnel regarding patient blood management [11, 12]. Therefore, as the second part of our study, we investigated whether an educational intervention of medical personnel could optimize filling volume of blood culture bottles. Finally, we assessed whether this reduction of blood volume had an impact on overall positive blood culture samples and on the time until the first positive blood culture.

## Methods

### Study design

This uncontrolled before and after study was performed as part of quality control and implementation within the framework of the patient blood management program at the General Hospital of the Medical University of Vienna, a 1800-bed tertiary hospital. Patient blood management was officially implemented in clinical routine of our institution starting in 2014. Approval to perform the study was obtained from the ethics committee of the Medical University of Vienna (#1664/2016). As only blood specimens sampled for routine clinical diagnostics were studied, the requirement for written informed consent to perform the study was waived by the ethics committee of the Medical University of Vienna. However, all individuals provided informed consent for blood to be withdrawn for clinical testing. Samples from minors were not included in the study. The study was performed following guidelines defined by the Declaration of Helsinki. All methods were carried out in accordance with relevant guidelines and regulations.

The first data acquisition phase lasted 4 months (May 2017–August 2017), immediately followed by the educational intervention (September 2017–February 2018). After the educational intervention, the second data acquisition phase again lasted for 4 months (March 2018–June 2018). Approximately 40,000 blood culture bottles are analyzed annually at our institution. We chose 4-month data acquisition periods to ensure inclusion of more than 5000 aerobic and 5000 anaerobic samples during each sampling phase. As we had no data regarding filling volume of blood culture bottles in a routine clinical setting, a sample size calculation could not be performed.

### Study-dependent variables

The primary outcome measure was the filling volume of blood culture bottles (BacT/Alert FA and FN; bioMérieux, Durham, NC, USA). According to the manufacturer’s instructions, the optimal filling volume for each individual blood culture bottle is 5–10 ml. We assessed the number of correctly filled bottles during both data acquisition phases. Secondary outcome measures were overall positive blood culture samples and the time until the first positive blood culture.

### Data collection

Blood culture bottles received by the microbiology diagnostics laboratory were weighed using an electronic precision scale (440-45 N; Kern, Balingen-Frommern, Germany). Blood volume withdrawn into each bottle was determined by subtracting the average weight of ten empty blood culture bottles from the measured weight.

### Microbiology testing

Blood cultures were loaded into BacT/Alert instruments and incubated for a maximum of 7 days. Whenever bottles were flagged as positive by the system, blood culture medium (1 ml) was harvested for direct identification using matrix-assisted laser desorption ionization-time of flight mass spectrometry-based technology (Bruker, Bremen, Germany), and subcultured for subsequent confirmation of identification.

### Educational intervention

All medical individuals involved in blood draws for blood culture testing at our hospital (physicians, nurses, medical technicians) were targeted by the educational intervention. One member of the medical study team held one 15-min lecture at each general ward and intensive care unit involved in blood culture testing. The medical study team consisted of anesthesiologists, intensive care physicians, and laboratory medicine physicians, all involved in patient blood management. During the lectures, physicians, nurses, and medical technicians were instructed regarding correct filling volume and handling of blood culture bottles. After the lectures, the study team distributed handouts describing the correct filling technique. The head nurse of each general ward or intensive care unit was instructed to inform team members not present during the lectures. In addition, short lectures (5–10 min) were held during grand rounds of surgical and medical disciplines.

As the study was performed within the framework of the patient blood management program at the General Hospital of the Medical University of Vienna, informational material was also circulated via email through the hospital’s mailing system. Finally, posters explaining correct filling information were placed at strategic places, such as individual storage spaces of blood culture bottles. Thus, whenever blood culture bottles were retrieved for sampling, the individual in charge of preparing the blood draw would see the correct instructions.

### Statistical analysis

Statistical analyses were performed using Prism 6.0 (GraphPad Software, La Jolla, CA, USA) and SPSS Statistics 25 (IBM, Ammok, NY, USA). A D’Agostino-Pearson test was used to verify normal distribution. Normally distributed data are depicted as mean with standard deviation. Statistical significance was set at *P* < 0.05. All analyses were performed separately for aerobic and anaerobic blood culture bottles. Blood culture bottles were categorized according to their filling volumes into three groups: underfilled bottles (filling volumes < 5 ml), correctly filled bottles (filling volumes 5-10 ml) and overfilled bottles (filling volumes > 10 ml). T-tests with Welch’s correction were used to compare filling volumes in each group before and after the educational intervention. Chi^2^ analysis was performed to test the independence between the three filling groups before and after the educational intervention. Additionally, Chi^2^ analysis was performed to test the independence between the incidence of positive blood culture results prior to and after the educational intervention. Linear regression analyses were performed to test the time to first positive blood culture. In the univariate regression model, we set the positive result of blood culture as the independent variable, the time until blood cultures yielded positive results as the dependent variable.

The interobserver reliability of measurements was performed by two independent observers in 100 sequential blood culture bottles. To measure intraobserver reliability, a single observer repeated measurements on the same blood culture bottles 2 days after the first measurement. Intraobserver and interobserver reliabilities were assessed using intraclass correlation coefficients. The risk of bias was assessed by using the ROBINS-I tool (classification into “low” - “moderate” - “serious” - “critical” risk) [13].

## Results

### Assessment of the risk of bias

We weighed 10,147 blood culture samples before and 11,806 blood culture samples after the educational intervention. Approximately 800 and 300 blood culture samples were not weighed during the first and second data acquisition periods, respectively (scheduling conflicts of the study team). Both measurements and time frame during which measurements were performed were standardized. The intraclass correlation coefficients for intraobserver and interobserver reliability of measuring the filling volume of blood culture bottles were 0.99. The risk of bias for non-randomized studies as assessed by the ROBINS-I tool was low to moderate. A detailed bias analysis is depicted in Table 1.

**Table 1:** Risk of bias as assessed by the ROBINS-I tool

Bias	Risk	Explanation
**Confounding**	Moderate	No control group; even though unlikely, the effect observed could occur without educational intervention
**Selection of study participants**	Moderate	Approximately 75% of possible participants received the educational intervention; effect could have been even larger when all health care professionals had received the educational intervention
**Classification of interventions**	Low	No co-interventions; intervention status is well defined and intervention definition is based solely on information collected at the time of intervention
**Deviations from intended interventions**	Low	No deviations from intended interventions and no co-interventions
**Missing data**	Low	Data are reasonably complete
**Measurement of outcomes**	Low	Outcome measures (filling volume of blood culture bottles and microbiology testing) are objective
**Selection of the reported results**	Low	Data are reported as planned

### Healthcare professionals receiving the educational intervention

At our institution, around 1500 healthcare professionals are involved in blood culture sampling. Approximately 60 % of these healthcare professionals attended one educational lecture at general wards, intensive care units, or during grand rounds. Of these not in attendance, another 40% of healthcare professionals were instructed by head nurses. Consequently, a total of about 75% of healthcare professionals involved in blood culture sampling were instructed during a lecture. An email with information regarding correct filling volume and technique was sent to all healthcare professionals through the hospital’s mailing system (Supplementary file).

### Educational intervention optimizes filling volume of blood culture bottles

**Fig. 1:**
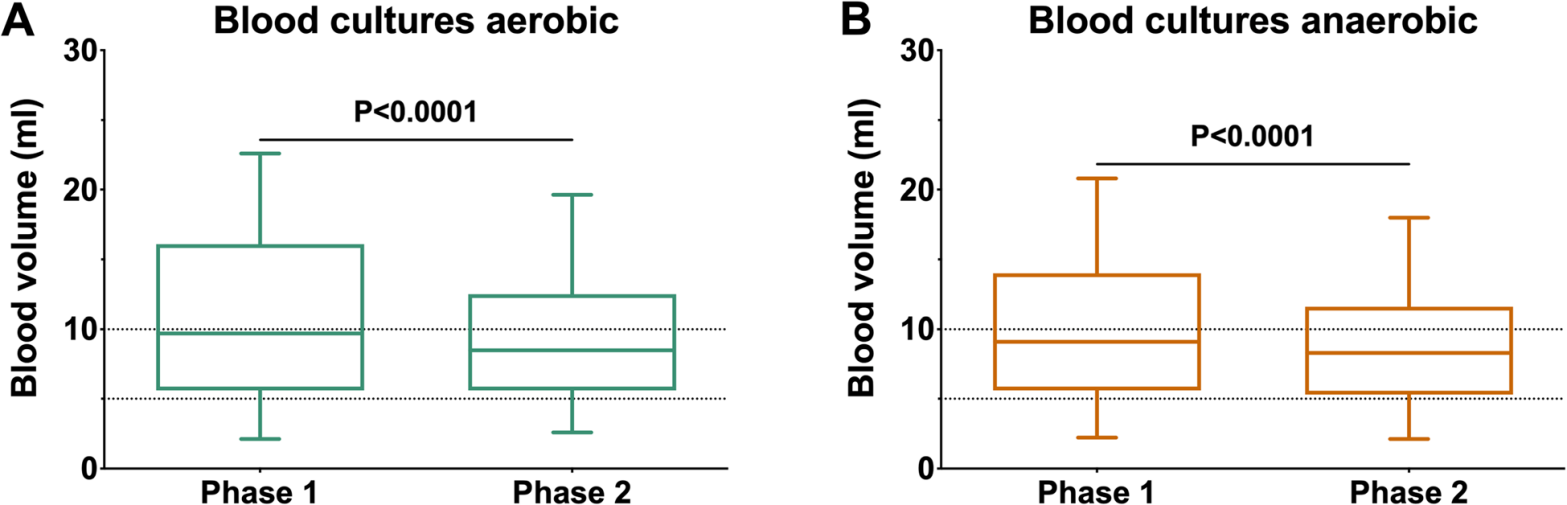
Distribution of blood volume in **a** aerobic and **b** anaerobic blood culture bottles before (Phase 1) and after (Phase 2) the educational intervention. Data in (**a**) and (**b**) are depicted as median with interquartile range and 95% confidence interval

The educational intervention increased the percentage of correctly filled blood culture bottles to 43% (*P* < 0.001) for both aerobic and anaerobic samples (Table 2). This increase in correctly filled bottles was accompanied by a reduction of bottles filled with > 10 ml of blood (*P* < 0.001). In addition, the educational intervention reduced withdrawn blood volume of aerobic samples from 11.0 ± 6.5 to 9.4 ± 5.1 ml (*P* < 0.001, Fig. 1a) and that of anaerobic samples from 10.1 ± 5.6 to 8.8 ± 4.8 ml (*P* < 0.001, Fig. 1b).

**Table 2:** Comparison of correctly and incorrectly filled blood culture bottles according to study periods

	Before education		After education		
Aerobic	n	%	n	%	*P* value
< 5 ml	1046	21	1092	19	0.62
*5–10 ml*	*1559*	*31*	*2478*	*43*	*< 0.001*
> 10 ml	2450	48	2243	39	< 0.001
Total	5055	100	5813	100	
Pearson Chi^2^ = 118, degree of freedom = 1					
**Anaerobic**	**n**	**%**	**n**	**%**	**P value**
< 5 ml	1042	20	1328	22	0.55
*5–10 ml*	*1747*	*34*	*2576*	*43*	*< 0.001*
> 10 ml	2303	45	2089	35	< 0.001
Total	5092	100	5993	100	
Pearson Chi^2^ = 131, degree of freedom = 1					

Correctly filled blood culture bottles (5–10 ml) are indicated by italic font

### Microbiological testing is not affected by reduced blood volume

**Fig. 2:**
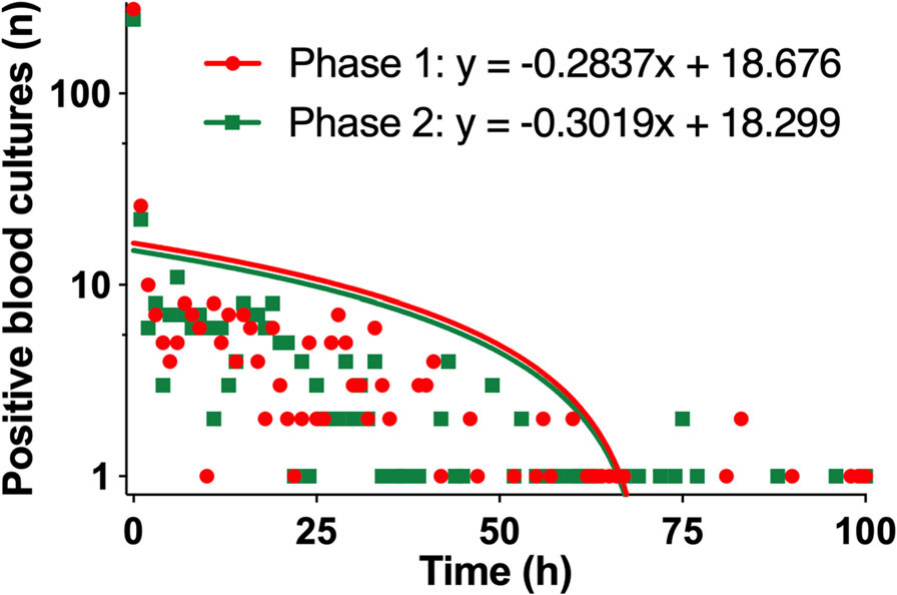
Time until blood cultures yielded positive results before (Phase 1, red dots) and after (Phase 2, green squares) the educational intervention. Dots and squares represent the number of individual blood cultures that yielded positive results at indicated time points (rounded up to each full hour). The y-axis is depicted logarithmically

Blood cultures were sampled and analyzed from 2266 patients before the educational intervention (Phase 1) and from 2362 patients after the educational intervention (Phase 2). In Phase 1, 493 patients (22%) had at least one positive sample, while 541 patients (23%) had at least one positive sample in Phase 2 (*P* = 0.35). Similarly, time to first positive blood culture did not differ among study periods (4.8 [95% CI 0.6–10.3] hours in Phase 1 vs. 4.5 [95% CI 0.3–9.3] hours in Phase 2, *P* = 0.87, Fig. 2).

## Discussion

In the current study, we evaluated the effect of an educational intervention on the filling volume of blood culture bottles. We report that the intervention increased the percentage of correctly filled bottles and reduced sample volume without having a negative effect on microbiology testing.

Closed blood sampling devices, implementation of point of care laboratory testing, and the use of small volume tubes were shown to reduce blood loss and requirement of blood transfusion in critically ill patients [14, 15]. Thus, implementation of multiple measures to reduce blood loss in hospitalized patients is crucial to save patients’ blood and prevent iatrogenic anemia. In our study, 1.5 ml blood less were withdrawn on average per sample after the educational intervention. Although 1.5 ml blood is a relatively small amount, extrapolating this figure to our hospital, where approximately 40,000 blood cultures are analyzed annually, 60 l of patients’ blood could be saved per year by a simple educational measure.

Looking at an individual patient basis, the International Guidelines for Management of Sepsis and Septic Shock recommend that two or more sets of blood cultures be obtained before initiation of antimicrobial therapy in patients with suspected sepsis [16]. When not disconnecting samples in time, 20 ml of blood can be withdrawn in excess. This practice would result in 80 ml of blood unnecessarily withdrawn with every two sets of blood cultures. As some patients are repeatedly tested, overfilling of blood culture bottles could quickly lead to significant blood loss. Therefore, adhering to correct filling volume could result in diminished blood loss and potentially fewer blood transfusions.

Positive blood culture rates were similar before and after the educational intervention. Thus, the smaller blood sample volume used for microbiology testing did not negatively affect microbiology results. These results are in line with a previous study assessing the impact of small volume tubes on routine laboratory testing [17]. In contrast, Khare et al. recently reported a vast majority (63.2%) of underfilled bottles in 10 hospitals in the greater New York Area [10]. Filling volume of more than 500,000 blood culture bottles was measured using automated systems. Interestingly, only 2.6% of blood culture bottles were overfilled during the baseline phase, completely opposite to the results observed in our study. An intervention phase resulted in a marked increase of correctly filled bottles and a greater rate of positive culture results. These differences among studies could be explained by different regional clinical settings or by the different blood culture systems used in both studies.

In general, educational interventions in the field of patient blood management have been shown to be effective, although the effect often disappears over time unless additional interventions are performed to sustain improvement. Vaghar reported an increase in nurses’ level of knowledge about the risks of blood transfusion after an educational intervention [12]. Chau et al. implemented a multidisciplinary patient blood management program at a vascular unit [18]. The implementation involved educational lectures, consultations, and discussions with doctors, nurses, and theater staff. Before implementation, 37% of patients received blood transfusions, whereas only 20% of patients were transfused in the follow-up phase.

Our study acts as an appeal to educate medical personnel regarding patient blood management. Anemia is a medical condition affecting more than 30% of the global population [19]. Even though patient blood management is associated with a reduction of red blood cell use without negatively affecting patient safety [2], major deficits exist in the implementation of patient blood management among clinicians [20]. Simple measures such as minimizing blood loss by using small-volume sampling tubes or preventing excess blood loss during blood draws could potentially have large clinical effects. These aspects hold especially true during the COVID-19 pandemic. Healthcare systems are at or over their limit, and every educational intervention could compound into medical or financial relief [21]. It is also important to note that our results only apply to blood culture sampling in adults, as volumes withdrawn in pediatric patients might differ depending on the system used.

The study has several limitations. As we only assessed the filling status of blood culture bottles, we did not measure cumulative blood loss in individual patients. Although the educational measure reduced the filling volume of blood culture bottles, we do not know whether this optimization translates into reduced transfusion requirements. In addition, we did not investigate whether the improved filling status of blood culture bottles was a temporary or lasting effect. Future quality improvement projects aimed at filling volumes of blood culture bottles should also study long-term effects and integrate measures to solidify positive effects obtained by educational interventions. A study design with a longer follow-up including multiple post-implementation periods (e.g. monthly or quarterly analysis of filling volumes) [10] could have increased the robustness of the data as compared to the simple before and after study design used in our project. Furthermore, we cannot answer whether the effects of this educational measure also translate into improved outcomes in hospitalized patients. Since this is an uncontrolled before and after study, the effect observed - even though unlikely - could have also occurred without an educational intervention.

As worldwide efforts increase to prevent unnecessary blood loss, education of medical personnel is an effective way to save patients’ blood. These educational measures can reduce iatrogenic blood loss and could possibly moderate the extent of phlebotomy-induced anemia.

## Supplementary Information

**Additional file 1.**

## Data Availability

The datasets generated and/or analyzed during the current study are not publicly available due to Austrian Legislation, but are available from the corresponding author on reasonable request.
