# Supplementary material for: Education of medical personnel optimizes filling volume of blood culture bottles without negatively affecting microbiology testing
Source: BMC Health Serv Res. 2020 Dec 1;20:1105. doi: 10.1186/s12913-020-05959-z (PMC7704116; doi:10.1186/s12913-020-05959-z)
Supplement: Supplementary file 1 — Additional file 1. [file 12913_2020_5959_MOESM1_ESM.pdf]

BacT/Alert Blood Culture Bottles - Sampling Instructions

5-10 ml blood per bottle

„Old“ system
FN Plus

Filled

Empty

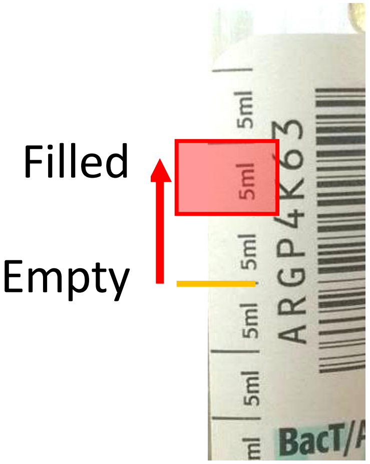

„New“ system
SA, SN, FA Plus

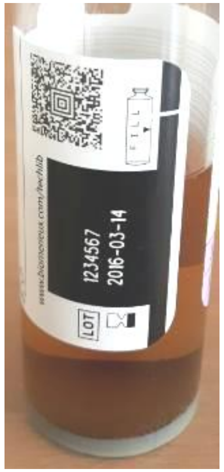

Filled

Empty

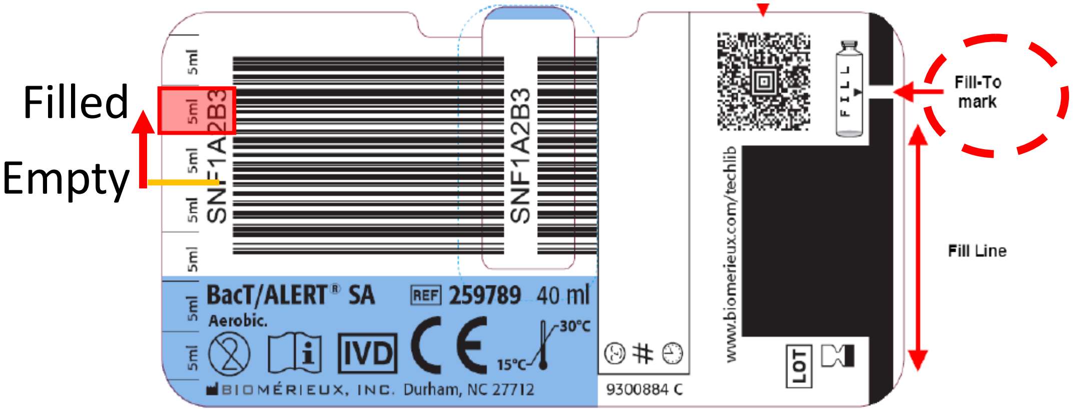

Marks are only valid when bottle is held upright.
